# Early-onset pancreatic neuroendocrine neoplasms: A distinct disease with improved survival compared with old individuals

**DOI:** 10.3389/fendo.2023.1025485

**Published:** 2023-04-04

**Authors:** Zhen Yang, Caiyun Liu, Kaiming Leng, Lianshuang Liu, Guangjun Shi

**Affiliations:** ^1^Department of Hepatopancreatobiliary Surgery, Qingdao Municipal Hospital, Qingdao University, Qingdao, China; ^2^Qingdao Hospital, University of Health and Rehabilitation Sciences (Qingdao Municipal Hospital), Qingdao, China; ^3^Department of Infectious Diseases, Qingdao Women and Children’s Hospital, Qingdao University, Qingdao, China

**Keywords:** early-onset pancreatic neuroendocrine neoplasms, later-onset pancreatic neuroendocrine neoplasms, incidence, clinical characteristics, survival

## Abstract

**Background:**

The incidence, clinicopathologic characteristics, treatment patterns, and survival of early-onset pancreatic neuroendocrine neoplasms (EOPanNENs) have not been well explored.

**Methods:**

Patients diagnosed with PanNENs were identified from the SEER database between 2000 and 2018. EOPanNENs were defined as diagnosis in patients aged less than 50 years, while the remaining were defined as later-onset pancreatic neuroendocrine neoplasms (LOPanNENs). Incidence, clinical features, management, and prognosis were analyzed in our study. Multivariable analyses were performed to identify factors associated with overall survival (OS) in EOPanNENs and LOPanNENs, respectively.

**Results:**

A total of 5172 patients with PanNENs were included: 1267 (24.5%) in the EOPanNENs cohort and 3905 (75.5%) in the LOPanNENs cohort. The age-adjusted incidence rate significantly increased among later-onset cases, while it remained relatively stable in early-onset cases. EOPanNENs were more frequently to be female, unmarried, and with better tumor differentiation compared with LOPanNENs. Of note, early-onset patients presented with a higher rate of lymph node involvement, and they were more likely to receive surgical treatment. For local-regional disease at presentation, surgery alone was the most frequently used regimen over the last two decades. With regard to distant stage, a combination of surgery and chemotherapy was more often utilized. Risk factors for PanNENs survival were more correlated with LOPanNENs compared with EOPanNENs. The OS and cancer-specific survival (CSS) were significantly better in the EOPanNENs group. Further analyses showed that EOPanNENs ≤ 2cm were associated with more favorable survival outcomes than EOPanNENs>2cm.

**Conclusion:**

EOPanNENs are a clinically rare and distinct entity from LOPanNENs. The advantages in survival for the EOPanNENs cohort over time were largely driven by the indolent clinical courses including better tumor differentiation and intensified surgical treatment. Further investigations are warranted to better understand the characteristics of this disease subgroup.

## Introduction

Pancreatic neuroendocrine neoplasms (PanNENs), originating from the diffuse endocrine system, are a heterogeneous group of uncommon epithelial tumors with diverse malignant potential (1, 2). Recent years the incidence of PanNENs has risen dramatically, which may primarily be attributed to routine screening and increased detection of asymptomatic disease (3–6). Although PanNENs typically affect elderly individuals, recent data indicate that the number of PanNENs in young adults aged less than 50 years old is steadily increasing. Previous studies focusing on other cancer types demonstrated significantly different epidemiologic characteristics and survival results between early-onset and later-onset cases, such as colorectal cancer (7–9). However, to the best of our knowledge, few large cohort studies have examined the epidemiology, risk factors, treatment patterns, and survival outcomes of patients with early-onset PanNENs (EOPanNENs) given the relative rarity and indolent clinical behaviors in comparison to pancreatic ductal adenocarcinoma (PDAC).

Therefore, the present study sought to systematically analyze and better define the incidence trends, clinical features, management strategies, and prognosis among patients with EOPanNENs over the last two decades using the information derived from a large population-based database in the United States.

## Methods

The Surveillance, Epidemiology, and End Results (SEER) program was used to identify patients who were pathologically diagnosed with primary PanNENs on the basis of conventional histology between 2000 and 2018: young age (<50 years), and older counterparts (≥50 years old). EOPanNENs were defined as diagnosis in patients aged less than 50 years of age, while the remaining were defined as later-onset pancreatic neuroendocrine neoplasms (LOPanNENs). The rationale for choosing 50 years old as the age threshold is not solely based on patient numbers, but rather on a combination of statistical considerations and clinical practice experience. Cancer is a complex disease that typically affects individuals aged 50 and above, but the increasing incidence of cancer in young adults under 50 suggests that there are changes in carcinogenic exposures that warrant attention. As mentioned, early-onset cancers typically present distinct pathological and biological features compared with later-onset cases, with these features more commonly observed in patients under 50 years old. Additionally, the age of 50 has been broadly accepted as the threshold for defining early-onset cancers in the medical community, enabling consistent comparisons between different studies and populations. The data on cancer epidemiology, clinicopathologic features, and survival outcomes were retrospectively collected and analyzed. Patients with missing data were not included in our study. The last follow-up time was December 31, 2018. The study was approved by the institutional review board (IRB) of Qingdao municipal hospital, and the informed consent was exempt for the data were obtained from a public database.

### Statistical analysis

Continuous data were presented as medians with interquartile ranges (IQR), and were compared using 2-tailed Student t-test. Categorical variables were expressed as number and percentage, and the differences between cohorts were examined by chi-squared test. And the survival outcomes including overall survival (OS) and cancer-specific survival (CSS) were estimated *via* Kaplan-Meier method with log-rank test. Univariate and multivariable Cox proportional hazard models were utilized to identify independent risk factors associated with OS for patients with EOPanNENs. Besides, the treatment distributions stratified by tumor stage (localized, regional, and distant) in the EOPanNENs cohort from 2000 to 2018 were assessed. All analyses were performed by SPSS 22.0 and R software, and a 2-sided P<0.05 was deemed to be statistically significant.

## Results

### Demographics and disease presentation

**Figure 1:**
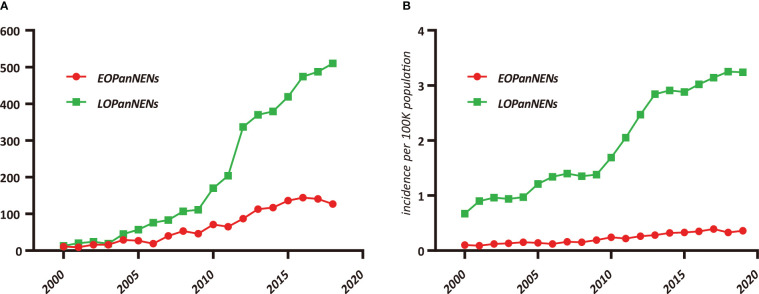
Trends in pancreatic neuroendocrine neoplasms (PanNENs) Incidence (2000–2018) in the United States, according to age. **(A)** Incidence of EOPanNENs and LOPanNENs cases per year. **(B)** Age-adjusted incidence of EOPanNENs and LOPanNENs in the overall population.

After inclusion and exclusion criteria were applied, a total of 5172 patients with PanNENs were identified and extracted from the SEER database between 2000 and 2018 for our study: 1267 (24.5%) with histologically confirmed LOPanNENs and 3905 (75.5%) with histologically confirmed LOPanNENs. Using population data from the SEER program, we calculated the annual number and the age-adjusted incidence of PanNENs cases during the study period, referring to the 2000 US standard population. Among LOPanNENs populations, the incidence rate significantly increased during the period from 2000 to 2018, whereas rate for EOPanNENs remained unchanged, as presented in **Figure 1**. Demographics, clinical characteristics, and survival outcomes of EOPanNENs vs LOPanNENs were summarized in **Table 1**.

**Table 1:** SEER cohort demographics, pathologic characteristics, and survival outcomes.

Variables	Overall (N=5172)	EOPanNENs (N=1267)	LOPanNENs (N=3905)	P value
Age, median (IQR)	60 (50 - 68)	42 (35 - 46)	64 (57 - 71)	
Gender				<0.001
Male	2779 (53.7%)	603 (47.6%)	2176 (55.7%)	
Female	2393 (46.3%)	664 (52.4%)	1729 (44.3%)	
Ethnicity				<0.001
White	4006 (77.5%)	923 (72.9%)	3083 (79.0%)	
Black	600 (11.6%)	169 (13.3%)	431 (11.0%)	
Other	566 (10.9%)	175 (13.8%)	391 (10.0%)	
Marital status				<0.001
Married	3249 (62.8%)	724 (57.1%)	2525 (64.7%)	
Other	1923 (37.2%)	543 (42.9%)	1380 (35.3%)	
Tumor size (cm), median (IQR)	3.0 (1.7 - 5.0)	3.0 (1.8 - 5.0)	3.0 (1.7 - 5.0)	0.846
Tumor grade				<0.001
Well differentiated	3575 (69.1%)	901 (71.1%)	2674 (68.5%)	
Moderately differentiated	1090 (21.1%)	280 (22.1%)	810 (20.7%)	
Poorly differentiated	507 (9.8%)	86 (6.8%)	421 (10.8%)	
Tumor number				<0.001
Single	4687 (90.6%)	1202 (94.9%)	3485 (89.2%)	
Multiple	485 (9.4%)	65 (5.1%)	420 (10.8%)	
Tumor location				0.037
Head	1543 (29.8%)	397 (31.3%)	1146 (29.3%)	
Body/Tail	2710 (52.4%)	625 (49.4%)	2085 (53.4%)	
Other	919 (17.8%)	245 (19.3%)	674 (17.3%)	
Functional status				0.172
Functional	430 (8.3%)	117 (9.2%)	313 (8.0%)	
Nonfunctional	4742 (91.7%)	1150 (90.8%)	3592 (92.0%)	
Lymph node involvement				0.025
Yes	1393 (26.9%)	372 (29.4%)	1021 (26.1%)	
No	3779 (73.1%)	895 (70.6%)	2884 (73.9%)	
Liver involvement				<0.001
Yes	792 (15.3%)	181 (14.3%)	611 (15.6%)	
No	3991 (77.2%)	950 (75.0%)	3041 (77.9%)	
Unknown	389 (7.5%)	136 (10.7%)	253 (6.5%)	
Tumor stage				0.055
Localized	2655 (51.3%)	619 (48.9%)	2036 (52.2%)	
Regional	1278 (24.7%)	343 (27.1%)	935 (23.9%)	
Distant	1239 (24.0%)	305 (24.0%)	934 (23.9%)	
Surgery				<0.001
Yes	3938 (76.1%)	1055 (83.3%)	2883 (73.8%)	
No	1234 (23.9%)	212 (16.7%)	1022 (26.2%)	
Radiation				0.737
Yes	184 (3.6%)	47 (3.7%)	137 (3.5%)	
No	4988 (96.4%)	1220 (96.3%)	3768 (96.5%)	
Chemotherapy				0.664
Yes	736 (14.2%)	185 (14.6%)	551 (14.1%)	
No	4436 (85.8%)	1082 (85.4%)	3354 (85.9%)	
Primary endpoint: OS, months				
Median (95% CI)	151.0 (138.0-164.0)	212.0 (186.7-237.3)	138.0 (125.8-150.2)	**<0.001†**

EOPanNENs, early-onset pancreatic neuroendocrine neoplasms; LOPanNENs, late-onset pancreatic neuroendocrine neoplasms; IQR, interquartile range; SEER, surveillance, epidemiology and end results; OS, overall survival; CI, confidence interval. †Log-rank test, Bold indicates significance.

**Figure 2:**
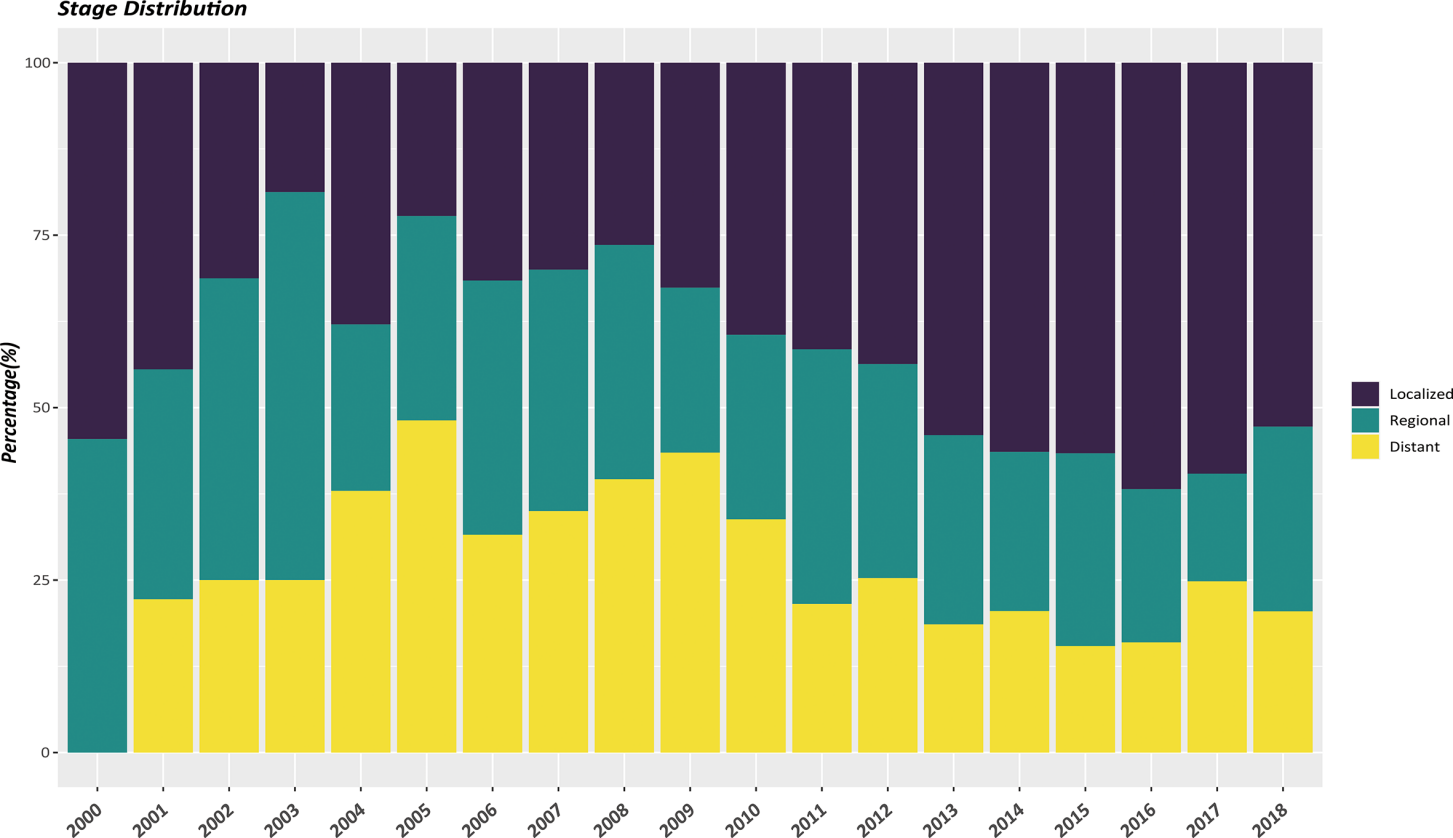
Shifts in stage at diagnosis among EOPanNENs patients in the United States, 2000-2018.

In the whole study population, a vast majority of patients (90.6%) had a solitary primary tumor while 9.4% had multiple tumors. Non-functional PanNENs patients accounted for approximately 90% of all the enrolled cases. As shown in **Table 1**, there were significant differences of patients’ characteristics among those two cohorts. EOPanNENs patients were more often female (52.4% vs 44.3%) and unmarried (42.9% vs 35.3%). Patients with EOPanNENs were also more frequently to have well to moderately differentiated histologic grade (92.2% vs 89.2%). Of note, compared to patients with LOPanNENs, those in the EOPanNENs cohort had a higher rate of lymph node involvement (29.4% vs 26.1%, P=0.025). Early detection and increasing public attention over the last few decades had led to population stage shift for PanNENs. As presented in **Figure 2**, the proportion of local-regional disease exhibited an obviously increasing trend among recent years. In terms of the management, patients with EOPanNENs were more likely to undergo surgical intervention (83.3% vs 73.8%). More detailed information on baseline characteristics were given in **Table 1**.

### Treatment distribution

**Figure 3:**
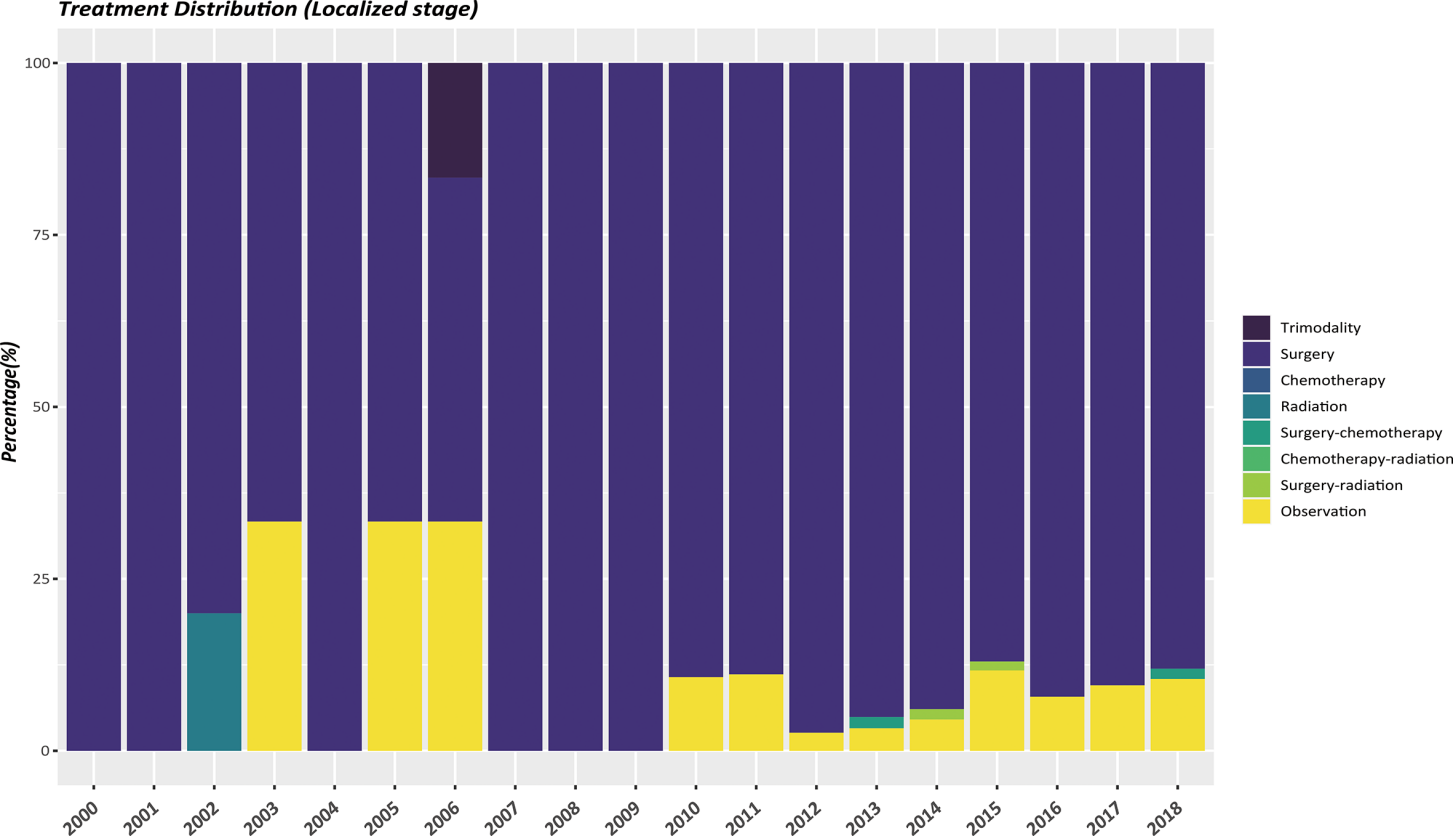
Differences in localized stage by type of treatment, 2000-2018, age<50.

**Figure 4:**
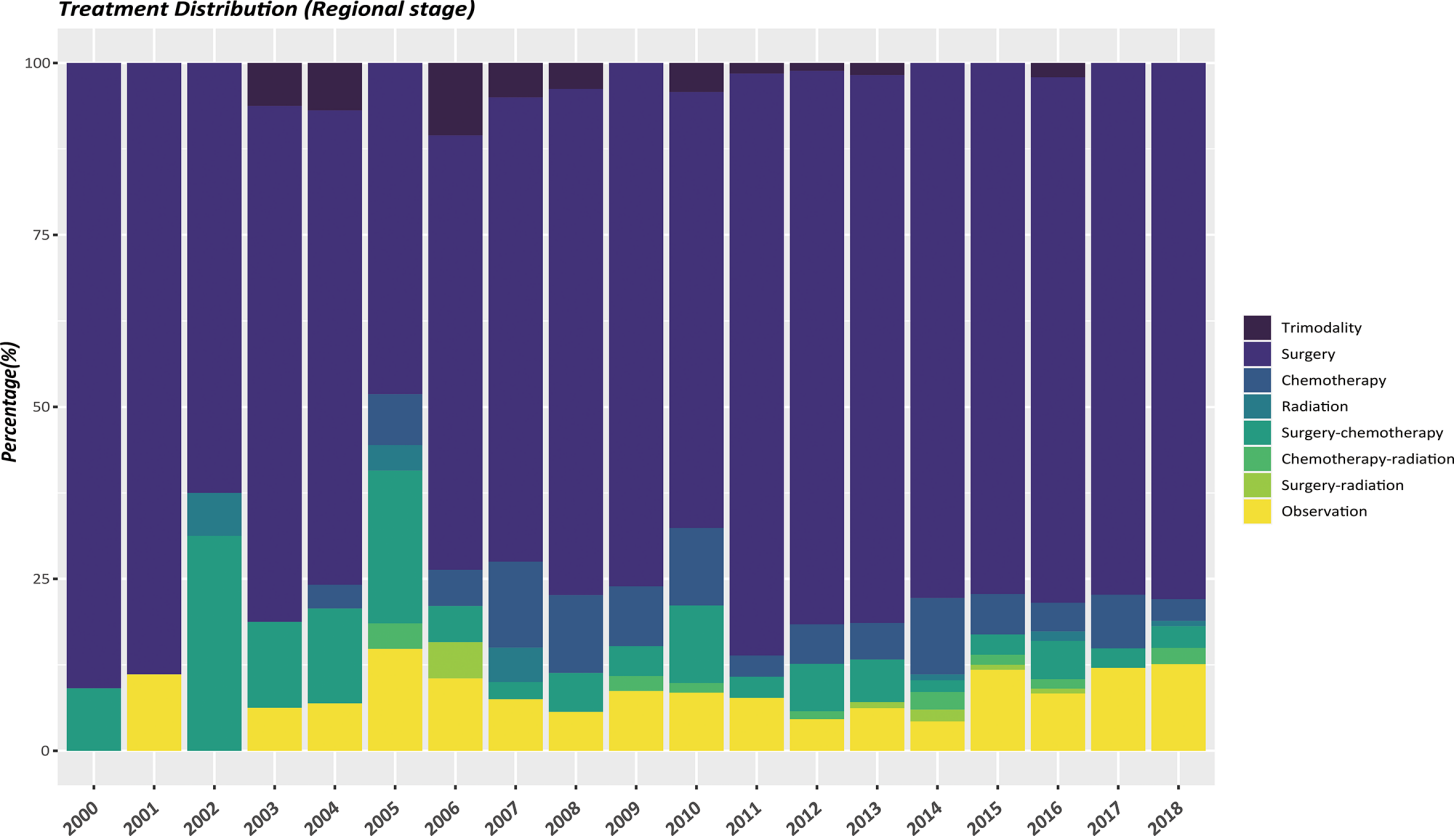
Differences in regional stage by type of treatment, 2000-2018, age<50.

**Figure 5:**
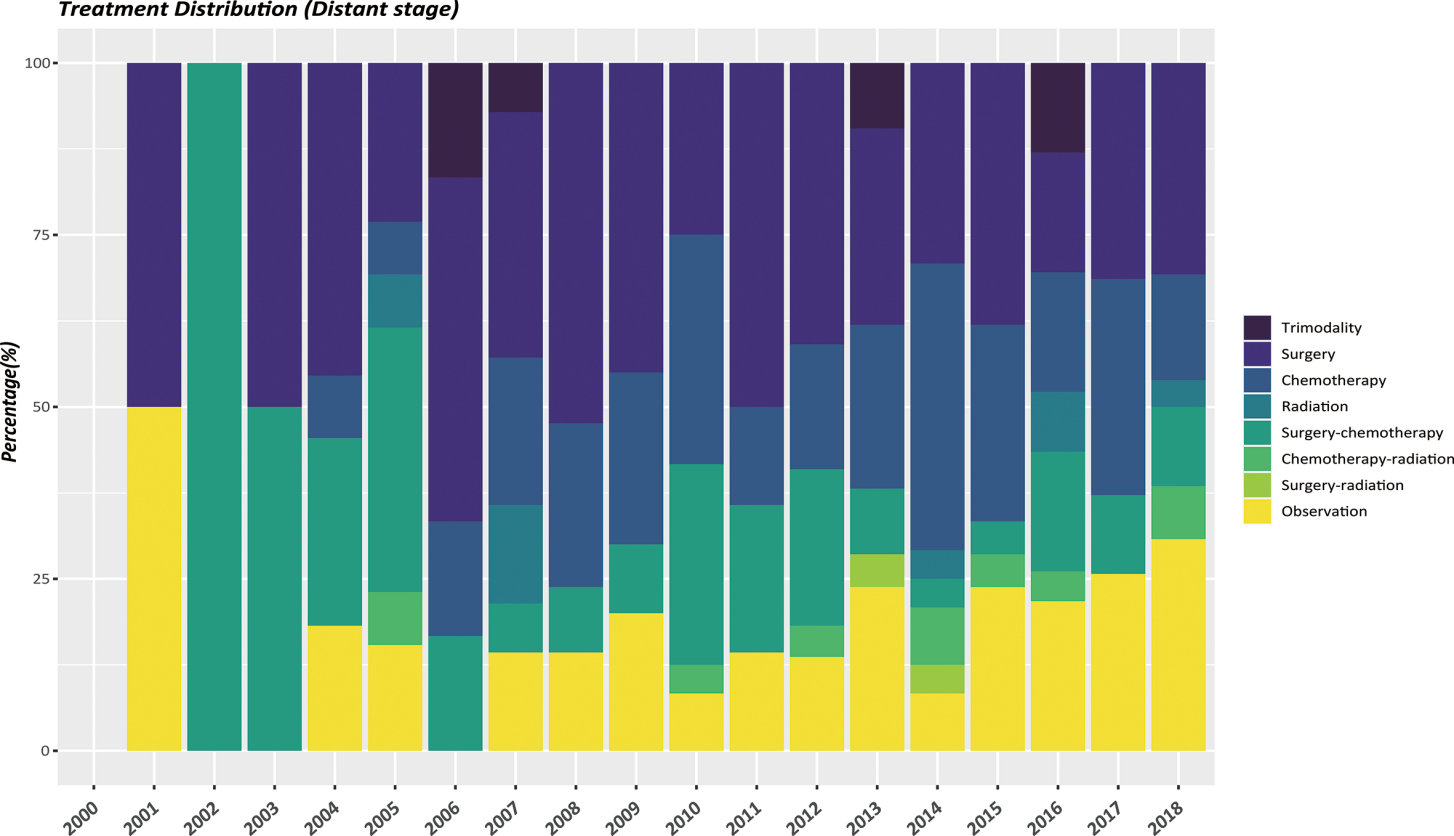
Differences in distant stage by type of treatment, 2000-2018, age<50.

The therapeutic modalities in the EOPanNENs cohort stratified by cancer stage (localized, regional, and distant) were then evaluated, respectively. For local-regional disease at presentation, surgical resection alone was the most frequently used regimen over the last two decades. (**Figures 3**, **4**) With regard to distant stage at presentation, a combination of surgery and chemotherapy was more often utilized among all years between 2000 and 2018 (**Figure 5**).

### Predictors of OS

Cox regression was then performed to select factors that best predicted prognosis of patients with PanNENs. For patients with EOPanNENs, univariate analysis yielded that gender, tumor size, tumor grade, lymph node involvement, tumor stage, surgery, chemotherapy, and radiation were associated with OS. In multivariable Cox proportional hazards model, poor differentiation, advanced tumor stage, and surgical resection were found to be with improved survival outcomes. As for LOPanNENs patients, multivariable survival analysis identified that gender, marital status, tumor size, histologic grade, tumor location, stage, and surgery were independent prognostic factors of OS (**Table 2**).

**Table 2:** Univariate and multivariate Cox regression analyses for factors affecting OS in patients with EOPanNENs or LOPanNENs.

Variables	EOPanNENs				LOPanNENs			
	Univariate analysis		Multivariate analysis		Univariate analysis		Multivariate analysis	
	HR (95% CI)	P value	HR (95% CI)	P value	HR (95% CI)	P value	HR (95% CI)	P value
Gender								
Male	Ref		Ref		Ref		Ref	
Female	0.66 (0.51, 0.85)	**0.002**	0.79 (0.60, 1.02)	0.073	0.76 (0.67, 0.87)	**<0.001**	0.83 (0.73,0.95)	**0.005**
Ethnicity								
White	Ref				Ref		Ref	
Black	1.24 (0.87, 1.78)	0.230			1.15 (0.95, 1.39)	0.158	0.99 (0.81, 1.20)	0.897
Other	0.79 (0.52, 1.21)	0.279			0.71 (0.55, 0.90)	**0.005**	0.79 (0.61, 1.01)	0.057
Marital status								
Married	Ref				Ref		Ref	
Other	1.19 (1.92, 1.55)	0.188			1.29 (1.14, 1.47)	**<0.001**	1.27 (1.11, 1.46)	**<0.001**
Tumor size								
≤2 cm	Ref		Ref		Ref		Ref	
>2 cm	3.07 (2.06, 4.60)	**<0.001**	1.25 (0.81, 1.93)	0.319	2.96 (2.49, 3.53)	**<0.001**	1.24 (1.01, 1.51)	**0.040**
Tumor grade								
Well differentiated	Ref		Ref		Ref		Ref	
Moderately differentiated	1.74 (1.27, 2.38)	**0.001**	1.35 (0.98, 1.85)	0.066	1.67 (1.42, 1.96)	**<0.001**	1.24 (1.05, 1.46)	**0.012**
Poorly differentiated	7.03 (5.10, 9.69)	**<0.001**	3.55 (2.50, 5.05)	**<0.001**	7.09 (6.13, 8.20)	**<0.001**	3.71 (3.13, 4.39)	**<0.001**
Tumor location								
Head	Ref				Ref		Ref	
Body/Tail	0.92 (0.68, 1.23)	0.565			0.58 (0.50, 0.66)	**<0.001**	0.80 (0.69, 0.93)	**0.003**
Other	1.01 (0.71, 1.44)	0.960			0.86 (0.73, 1.02)	0.084	0.89 (0.75, 1.06)	0.186
Functional status								
Non-Functional	Ref				Ref			
Functional	1.04 (0.64, 1.71)	0.871			0.88 (0.67, 1.16)	0.371		
Lymph node involvement								
No	Ref		Ref		Ref		Ref	
Yes	2.51 (1.94, 3.26)	**0.006**	1.28 (0.95, 1.74)	0.106	1.67 (1.47, 1.90)	**<0.001**	1.13 (0.97, 1.31)	0.119
Tumor stage								
Localized	Ref		Ref		Ref		Ref	
Regional	3.22 (2.08, 4.99)	**<0.001**	1.96 (1.18, 3.23)	**0.009**	2.07 (1.73, 2.48)	**<0.001**	1.48 (1.19, 1.85)	**0.001**
Distant	10.6 (7.11, 15.7)	**<0.001**	4.33 (2.63, 7.12)	**<0.001**	6.47 (5.53, 7.56)	**<0.001**	2.44 (1.97, 3.01)	**<0.001**
Surgery								
No	Ref		Ref		Ref		Ref	
Yes	0.19 (0.15, 0.25)	**<0.001**	0.41 (0.30, 0.58)	**<0.001**	0.17 (0.15, 0.20)	**<0.001**	0.28 (0.24, 0.32)	**<0.001**
Chemotherapy								
No	Ref			Ref		Ref	Ref	
Yes	5.38 (4.14, 7.00)	**<0.001**	1.38 (0.99, 1.92)	0.053	4.33 (3.79, 4.94)	**<0.001**	0.95 (0.81,1.12)	0.543
Radiation								
No	Ref		Ref		Ref		Ref	
Yes	4.30 (2.89,6.39)	**<0.001**	1.25 (0.81, 1.94)	0.317	2.85 2.28, 3.56)	**<0.001**	0.99 (0.79, 1.26)	0.953

OS, overall survival; EOPanNENs, early-onset pancreatic neuroendocrine neoplasms; LOPanNENs, late-onset pancreatic neuroendocrine neoplasms; HR, hazard ratio; CI, confidence index; Ref, reference. Bold indicates significance.

### Survival disparity between EOPanNENs and LOPanNENs

**Figure 6:**
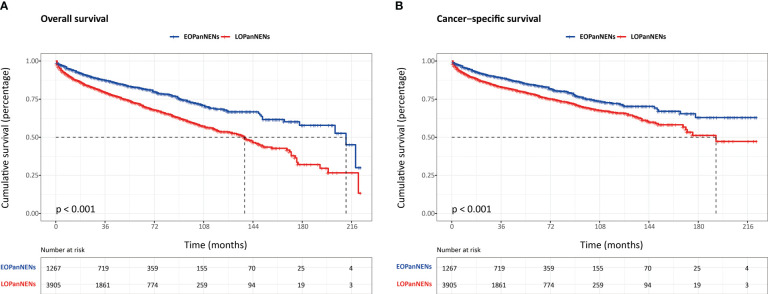
The overall survival **(A)** and cancer-specific survival **(B)** between EOPanNENs and LOPanNENs.

**Figure 7:**
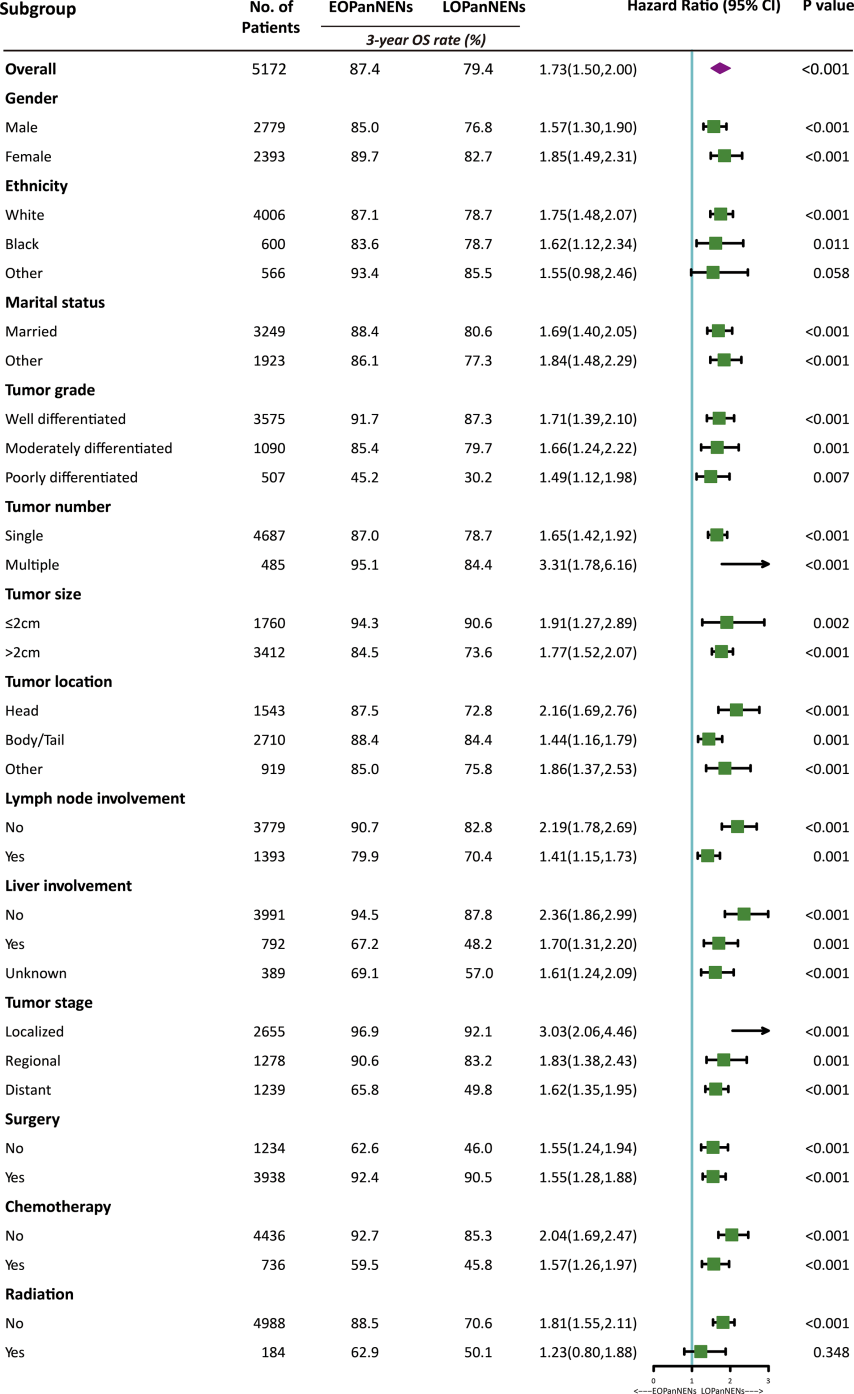
3-year overall survival of EOPanNENs compared with LOPanNENs in subgroups of patients with different tumor characteristics and treatment types.

**Figure 8:**
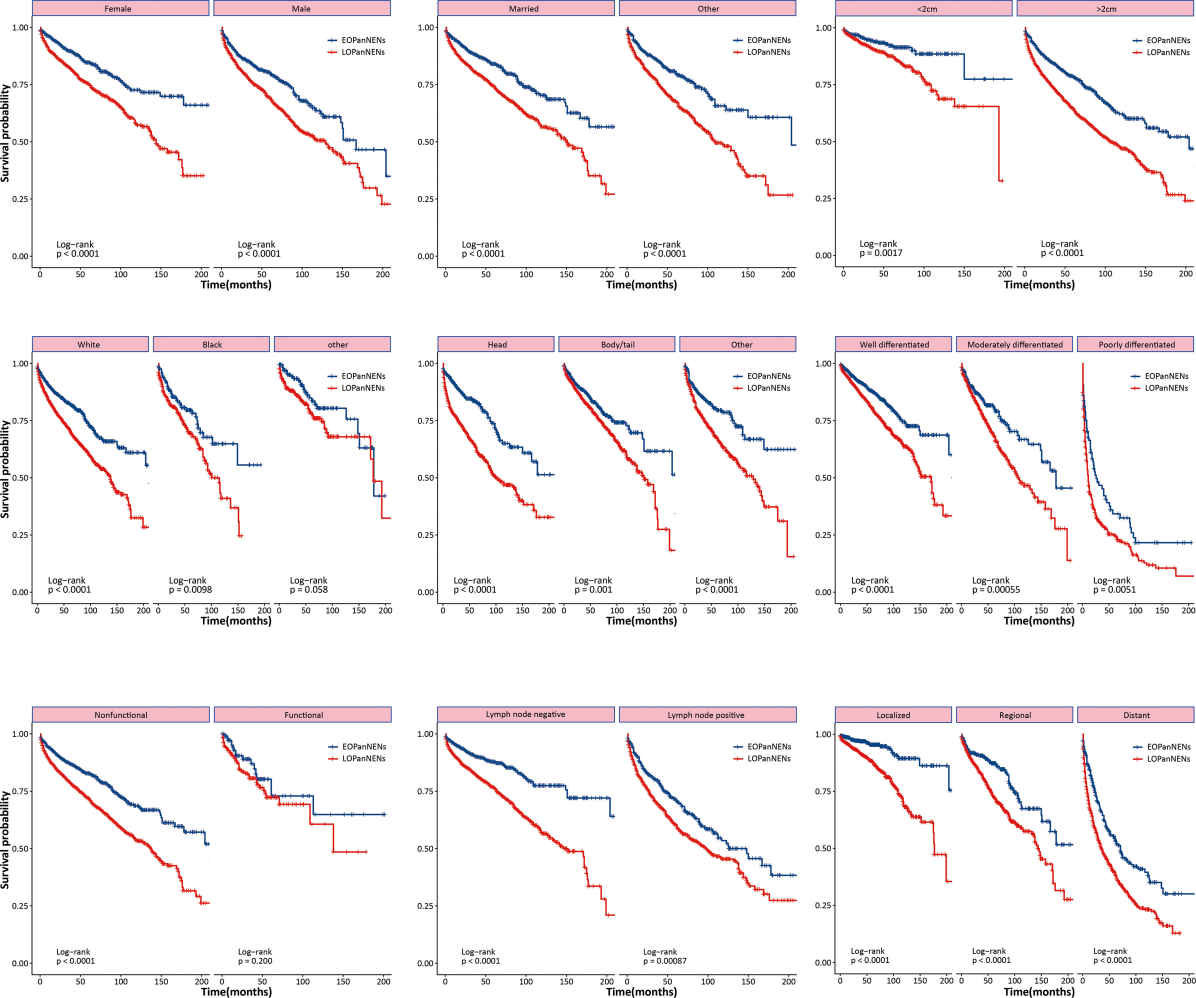
Subgroup analyses of overall survival between patients with EOPanNENs and LOPanNENs.

The overall survival (OS) and cancer-specific survival (CSS) of young adults were significantly better than that of older counterparts (**Figure 6**). The median OS was 212.0 months for patients with EOPanNENs, while 138.0 months for those with LOPanNENs. In addition, the 3-year OS between EOPanNENs and LOPanNENs in all prespecified subgroups was then assessed in our survival analyses (**Figures 7**, **8**). Cases with EOPanNENs were associated with a significantly better 3-year OS compared with LOPanNENs in all these subgroups except for those with other ethnicity or those who underwent radiation. Surely, patients who received radiation were more likely to have a higher tumor burden and more aggressive tumor biology.

### Exploratory analyses

**Figure 9:**
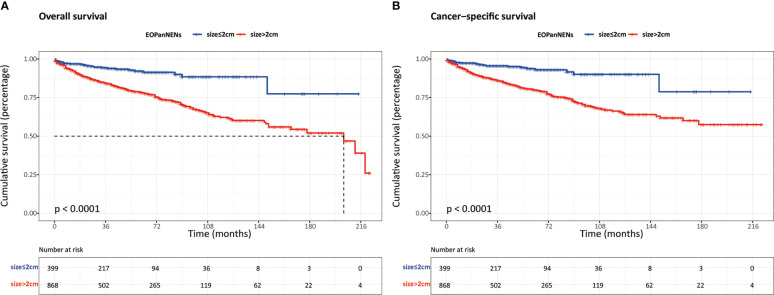
The overall survival **(A)** and cancer-specific survival **(B)** between EOPanNENs ≤2cm and EOPanNENs >2cm.

In order to better define the impact of tumor size on survival outcomes in EOPanNENs, we analyzed the clinical characteristics and survival between patients with EOPanNENs ≤ 2cm and those with EOPanNENs>2cm. As shown in the **Table 3**, the baseline characteristics were significantly different among these two cohorts. Kaplan-Meier curves indicated that the OS and CSS were more favorable in patients with EOPanNENs ≤ 2cm, as compared with that in patients with EOPanNENs >2cm (**Figure 9**).

**Table 3:** Patient characteristics of EOPanNENs ≤2 cm versus >2 cm in the SEER database.

Variables	EOPanNENs ≤2 cm (N=399)	LOPanNENs >2 cm (N=868)	P value
Gender			0.001
Male	162 (40.6%)	441 (50.8%)	
Female	237 (59.4%)	427 (49.2%)	
Ethnicity			0.658
White	284 (71.2%)	639 (73.6%)	
Black	56 (14.0%)	113 (13.0%)	
Other	59 (14.8%)	116 (13.4%)	
Marital status			0.903
Married	229 (57.4%)	495 (57.0%)	
Other	170 (42.6%)	373 (43.0%)	
Tumor grade			<0.001
Well differentiated	322 (80.7%)	579 (66.7%)	
Moderately differentiated	60 (15.0%)	220 (25.3%)	
Poorly differentiated	17 (4.3%)	69 (7.9%)	
Tumor number			0.687
Single	380 (95.2%)	822 (94.7%)	
Multiple	19 (4.8%)	46 (5.3%)	
Tumor location			0.871
Head	121 (30.3%)	276 (31.8%)	
Body/Tail	200 (50.1%)	425 (49.0%)	
Other	78 (19.5%)	167 (19.2%)	
Functional status			0.153
Functional	30 (7.5%)	87 (10.0%)	
Nonfunctional	369 (92.5%)	781 (90.0%)	
Lymph node involvement			<0.001
Yes	47 (11.8%)	325 (37.4%)	
No	352 (88.2%)	543 (62.6%)	
Liver involvement			<0.001
Yes	20 (5.0%)	161 (18.5%)	
No	364 (91.2%)	586 (67.6%)	
Unknown	15 (3.8%)	121 (13.9%)	
Tumor stage			<0.001
Localized	315 (78.9%)	304 (35.0%)	
Regional	52 (13.0%)	291 (33.5%)	
Distant	32 (8.0%)	273 (31.5%)	

EOPanNENs, early-onset pancreatic neuroendocrine neoplasms; SEER, surveillance; epidemiology and end results; CI, confidence interval. Bold indicates significance.

## Discussion

In this comprehensive study of pancreatic neuroendocrine neoplasms (PanNENs), our population-based analysis found a steady rise of the incidence of LOPanNENs in the United States over last two decades, whereas the incidence of EOPanNENs remained relatively stable. And we further investigated the differences between EOPanNENs and LOPanNENs using the largest cohort of PanNENs cases reported as yet, with a focus on epidemiology, clinicopathologic characteristics, and survival outcomes. The findings of our study suggested that patients with EOPanNENs were associated with distinct clinical features and prognosis in comparison to those with LOPanNENs. Results from subgroup analyses further indicated that PanNENs survival was generally better for patients diagnosed before 50 years old. Additionally, our analysis showed that a high percentage of patients with EOPanNENs were diagnosed as having lymph node involvement. Surgery remained the most frequently utilized therapy in cases with local-regional disease, while those with distant disease were more likely to be treated with a combination of surgery and chemotherapy.

The incidence of PanNENs is projected to steadily increase, likely attributable to the high-resolution imaging and increased utility of diagnostic techniques (10, 11). A large proportion of neoplasms were diagnosed incidentally during imaging conducted for an unrelated diagnosis (12). Our results showed that the number of LOPanNENs patients increased more pronounced than the EOPanNENs cases. With respect to the annual incidence, LOPanNENs patients experienced a faster increase than EOPanNENs patients. As shown in our study, the annual age-adjusted incidence of EOPanNENs remained unchanged, while a marked increase of LOPanNENs occurred in United States during the last two decades. The explanation for this phenomenon might in part be the enhanced availability of routine monitoring in elderly adults. Among stage groups, local-regional disease accounted for the majority of PanNENs, which might also mainly be caused by the rise in early detection capability (13). By the way, both of the EOPanNENs and LOPanNENs cohorts shared the similar stage distribution according to our study.

It is reported that PanNENs exhibited a slight male predominance (14). However, in our study, early-onset patients showed a female preponderance (52.4%) compared with men (47.6%). The exact cause of gender differences between EOPanNENs and LOPanNENs was not well-learned. Previous studies argued that the distribution of risk factors might play a role in the sex disparity (15, 16).

Patients with EOPanNENs in our study seemed to be associated with lower tumor burden and less aggressive behaviors compared to those with LOPanNENs, except for the higher rate of lymph node involvement. Delayed diagnosis in younger patients and presentation with more metastatic lymph nodes highlighted the necessity for the great awareness of the disease on general public, as well as the enhancement of detection ability. Younger patients were more frequently to receive surgical treatment, making it convenient to evaluate the lymph node status. And our study also demonstrated that early-onset patients had significantly better survival outcomes compared to later-onset cases despite more lymph node involvement of EOPanNENs.

Even though there existed numerous treatment options for PanNENs, surgery remained the cornerstone of treatment, which has been proved to be with survival benefits (17–20). Some studies even concluded that cancer-directed surgery can also provide improved survival outcomes in patients with distant diseases (21–25). Consistent with prior findings, we found that patients with EOPanNENs were more likely to complete surgical resections for the primary tumor, and the OS and CSS were significantly better in these patient population compared to LOPanNENs. As for metastatic patients, surgery and chemotherapy were more commonly be proposed as an adequate management as it conferred survival advantages in selected patients (26).

A significant difference in risk factors existed between EOPanNENs and EOPanNENs cohorts. Similar to other studies focusing on the whole PanNENs population, survival analyses using the SEER database confirmed previous results of the prognostic significance of gender, tumor diameter, tumor differentiation, location, stage at presentation, and surgery for LOPanNENs (27, 28). While only poor differentiated tumors, advanced stage, and surgical intervention were significantly associated with OS in the patients with EOPanNENs, which again confirmed EOPanNENs as a unique clinical entity.

Our study has several limitations. First, given the retrospective nature, it is unlikely to avoid the selection biases. And the SEER database does not record novel medications and treatments that have been adopted to improve survival in patients with PanNENs. Second, information regarding to treatment regimens, perioperative complications, and disease recurrence were not available in the public data source, which may limit the generalization of the conclusion (29). However, such drawbacks are inevitable and inherent to any retrospective, population-based analysis. Furthermore, the dichotomy at 50 years of age has its limitations. While there are differences in epidemiology, clinicopathological, and molecular characteristics between early-onset and later-onset tumors, these features are less likely to change dramatically at precisely 50 years of age. We recognize the constraints of using a dichotomy at 50 years of age, but we chose this cutoff point to ensure consistent collection and interpretation of existing evidence on early-onset cancers. In reality, the heterogeneity within this group should also be taken into account. Considering the varying age distribution of cancer diagnosis by different organs, the optimal screening and treatment strategies for specific age groups should be tailored based on the specific organ site affected. The strength of our study compared to previous studies is the largest sample size of PanNENs patients utilized to characterize the clinicopathologic features and survival outcomes for the first time.

In conclusion, unlike the rapid increase in incidence rate of LOPanNENs patients, the age-adjusted incidence of EOPanNENs remained stable according to the analysis of SEER database between 2000 and 2018. Diagnoses of better tumor differentiation represented a larger proportion of the EOPanNENs cohort over the last two decades, together with the higher rate of surgical treatment, resulting in the more favorable survival outcomes compared to LOPanNENs.

## Data availability statement

The datasets presented in this study can be found in online repositories. The names of the repository/repositories and accession number(s) can be found below: https://seer.cancer.gov/.

## Author contributions

GS and LL contributed to the conception. CL and KL collected data. ZY and LL designed the study and wrote the manuscript. All authors contributed to the article and approved the submitted version.
